# The impact of glycemic status on radiological manifestations of pulmonary tuberculosis in diabetic patients

**DOI:** 10.1371/journal.pone.0179750

**Published:** 2017-06-19

**Authors:** Li-Kuo Huang, Hsueh-Han Wang, Yi-Chun Lai, Shi-Chuan Chang

**Affiliations:** 1 Department of Radiology, National Yang-Ming University Hospital, Yi-Lan, Taiwan; 2 School of Medicine, National Yang-Ming University, Taipei, Taiwan; 3 Department of Internal Medicine, Division of Chest Medicine, National Yang-Ming University Hospital, Yi-Lan, Taiwan; 4 Department of Chest Medicine, Taipei Veterans General Hospital, Taipei, Taiwan; 5 Institute of Emergency and Critical Care Medicine, National Yang-Ming University, Taipei, Taiwan; Karolinska Institutet, SWEDEN

## Abstract

**Setting:**

Diabetes mellitus (DM) may increase risk of pulmonary tuberculosis (PTB) and influence its radiological manifestations.

**Objective:**

To evaluate the impact of glycemic status on radiological findings of PTB in diabetic patients.

**Methods:**

Between January 2010 and December 2015, chest radiographs (CXRs) in consecutive 214 DM patients with culture-proved PTB and 123 available thoracic computed tomography (CT) scans were enrolled. An equal number of non-DM patients with similar demographics was included as the control group. Glycemic status was assessed by glycosylated hemoglobin (HbA1c), and a cutoff of 8% was used to further investigate radiological features of diabetic PTB. Two radiologists and one pulmonologist reviewed the chest images independently.

**Results:**

Compared with non-DM patients, primary PTB pattern and extensive disease on CXRs as well as primary PTB pattern, large non-cavitary nodule, more than one cavity in a single lesion, unusual location, and all lobe involvement of lesions on thoracic CT scans were more common in DM patients. Furthermore, diabetics with HbA1c > 8% were more likely to exhibit unusual findings (*P <* 0.001), far advanced extensive lesions (*P* < 0.001) on CXRs, lymphadenopathy (*P* = 0.028), more than one cavity in a single lesion (*P* < 0.001) and all lobe involvement (*P* = 0.041) on thoracic CT scans.

**Conclusions:**

Glycemic status influenced radiological manifestations of diabetic PTB. Given an increased risk of atypical radiological presentations of PTB in DM patients, physicians should be alert and pay more attention to those with poor glycemic control.

## Introduction

Tuberculosis (TB) and diabetes mellitus (DM) are both important global health issues. In addition, the link between TB and DM has been demonstrated, and these conditions may complicate each other at numerous levels. Concerning the epidemiology, approximately 70% of diabetics live in TB endemic countries [1], and the World Health Organization (WHO) reported that approximately 10% of TB cases worldwide are associated with DM [2]. In addition, the summary relative risk of TB in people with DM is 2.52 (95% CI: 1.53 to 4.03) in 4 cohort studies and 2.20 (ranged from 1.16 to 7.81) in 10 case-control studies [2,3]. In a cohort study by Leung et al, poor glycemic control was significantly associated with the occurrence of TB [4]. Another study from South Africa further demonstrated a correlation between active TB and the level of glycosylated hemoglobin (Hb_A1c_) with a hazard ratio of 1.39 (95% CI: 1.18–1.63) per unit increase [5].

In addition to an increased risk of developing TB, DM increases TB disease severity [6] and may lead to an increased risk of delayed sputum conversion, treatment failure, and recurrence/relapse after TB treatment [6,7]. Therefore, diabetic TB patients can play a critical role of TB transmission.

The effect of DM on the radiological manifestation of pulmonary TB (PTB) using chest radiographs (CXRs) was investigated over the past 20 years. These studies demonstrated that diabetic PTB patients might exhibit an increased frequency of atypical features, including lower lobe disease [8,9], less reticulonodular opacities [10], and more extensive disease [9,11–13]. In contrast, several studies revealed no obvious radiological differences between diabetic and non-diabetic PTB patients [10,14–16]. A relatively small number of study cases, inconsistent consideration of the influence of age and gender and the effect of different glycemic control levels may partially explain the mixed results of these reported studies. In 2014, a study with a larger sample size enrolling 581 culture-positive PTB cases with DM and similar number of non-DM PTB cases by Chiang et al. demonstrated an increased frequency of lower lung field opacity and extensive parenchymal involvement in diabetic PTB patients [17]. Moreover, this study indicated that the risk of lower lung field disease is potentially correlated with glycemic control in DM.

On the other hand, the CT findings of PTB in diabetic and non-diabetic cases were only investigated in one study with a small number of cases (31 DM cases, 71 cases without underlying disease) by Ikezoe and colleagues [18]. The results revealed an increased prevalence of non-segmental distribution of lesions in DM patients, but no significant difference in disease location was noted compared with the controls. To the best of our knowledge, no study has investigated the relation between glycemic status and CT findings of PTB diabetic patients.

DM may exhibit a risk for PTB and impact radiological manifestations of PTB in diabetics. In this study, we intended to evaluate the differences of CXRs and thoracic CT findings of PTB between diabetics and non-diabetics and further address the impact of glycemic status on imaging features of PTB in DM patients.

## Materials and methods

### Patients

This retrospective study was conducted from January 1, 2010 to December 21, 2015 and was approved by the Institutional of Review Board of National Yan-Ming University Hospital (NYMUH IRB No.2016A016). The medical records of all patients with positive cultures for *Mycobacterium tuberculosis* from respiratory specimens (sputum, bronchoalveolar lavage, lung aspiration, or lung biopsy) were eligible for this study, and only patients with pretreatment CXRs corresponding to the positive cultures were included in the study. Patients with extra-thoracic TB, human immunodeficiency virus (HIV) infection, undergoing immunosuppressant treatment, underlying malignancy, and concurrent pulmonary diseases, including lung cancer, pneumoconiosis and other pulmonary infections, were excluded from the study.

Further, DM cases were defined only in those 1) treated with insulin or diabetes-specific hypoglycemic agents, 2) assigned an ICD-9 code related to DM twice or more on outpatient visits or admission, or 3) had a fasting blood glucose concentration greater than 126 mg/dL at 2 different time points. For further evaluation of the association between glycemic status and radiological manifestations, only DM patients with glycosylated hemoglobin (Hb_A1C_) recorded within 3 months of the initiation of TB treatment were included in the TB DM group. Given that the majority of diabetic PTB patients were adults greater than 65 years of age in this study, a cutoff Hb_A1c_ of 8% was selected for glycemic status evaluation according to the suggestion of geriatric DM controls from a previous report [19]. Accordingly, TB DM patients were further divided into 2 subgroups: controlled with Hb_A1C_ ≤ 8% and uncontrolled with Hb_A1C_ > 8%.

An equal number of age- and gender-matched PTB cases diagnosed in the same period and without a history of DM, immunosuppressive treatment, known causes of immunosuppression and concurrent pulmonary diseases was randomly selected to serve as controls (TB group).

### Radiographic evaluation

Two radiologists (LKH, HHW) and one pulmonologist (YCL) who were blinded to the patients’ clinical information independently reviewed and interpreted the CXRs and thoracic CT scans using a standardized form. Final decision on the findings was achieved by consensus of at least two physicians. The cases of disagreement were presented to the conference consisting of the 3 readers and one senior expert pulmonologist (SCC, in practice for 36 years) for discussion, and the adjudicated reading after consensus was used as the final result.

#### Chest radiography

Abnormal PTB findings on CXRs were classified as ‘usual’ and ‘unusual’ patterns according to previous reports [20–24]. The usual pattern suggestive of post-primary PTB included fibronodular lesions without cavity; patchy heterogeneous consolidation mainly involving segments (S)1, S2 and S6; bronchogenic spreading; and tuberculoma with a range of 0.5 to 4.0 cm in diameter. Radiological findings indicative of an unusual pattern were as follows: 1) typical primary PTB, including lesions limited to lower lung fields (middle lobe, lingula and/or lower lobes), isolated hilar and/or mediastinal lymphadenopathy, pleural effusion alone, or more than one of the above findings; 2) other unusual findings, including miliary or disseminated lesions or negative finding on CXRs. The extent of radiographic disease was graded based on the U.S. National Tuberculosis and Respiratory Disease Association scheme that classified disease into minimal, moderately advanced, and far-advanced disease [25].

#### Thoracic CT scans

We analyzed the pattern and location of abnormalities on the thoracic CT scans [18,20,21,24]. The ‘usual’ pattern indicative of post-primary PTB included centrilobular nodules, branching linear and nodular opacities (tree-in-bud appearance), a single cavitary nodule, satellite nodules, and acinar or lobular nodules. The ‘unusual’ pattern consisted of the following: 1) primary PTB, including segmental or lobar consolidation, lymphadenopathy (lymph node with a short-axis diameter >10 mm) and pleural effusions; and 2) additional findings, including miliary lesions, pericardial effusion, single or multiple non-cavitary large nodules/masses greater than 1 cm, and more than one cavity in any single lesion.

Lesions were classified into three groups by site. Lesions were regarded as being in the usual location when they were limited to or mainly involved the apical and posterior segments of the upper lobes and the superior segments of the lower lobes, whereas lesions limited to or mainly involving the remaining segments of the lungs were considered unusual. Lesions were considered mixed when usual and unusual locations were evenly distributed.

### Statistical analysis

Comparisons of the categorical variables between the study and the control groups were performed using Fisher’s exact test. Comparisons of the continuous variables between the two groups were performed using the Mann-Whitney *U*-test. Statistical significance was defined as *P* < 0.05 (two-tailed). Statistical analysis was performed using SPSS v.15.0 (Statistical Product and Service Solutions, Chicago, IL, USA).

## Results

### Comparison of PTB in diabetics (TB DM group) and non-diabetics (TB group)

In total, 214 patients (172 men and 42 women) with culture-proved PTB and correlated CXRs were enrolled in both TBDM (age range 25–98 years, mean ± standard deviation (SD) 72.5 ± 15.4) and TB (age range 20–101 years, mean ± SD 72.4 ± 15.6) groups, and no significant differences were noted in age (*P* = 0.935). In addition, each of 123 cases (97 men and 26 women) with pretreatment thoracic CT scans at the diagnosis of PTB were selected to be included in both TBDM (age range 25–95 years, mean ± SD 70.3 ± 14.8) and TB (age range 33–95 years, mean ± SD 70.5 ± 14.6) groups. There was no significant difference in age between two groups (*P* = 0.934). Regarding smoking status, 102 cases in the TBDM group and 95 cases in the TB group were ever smokers, and no significant statistically differences were observed (*P* = 0.561).

**Fig 1:**
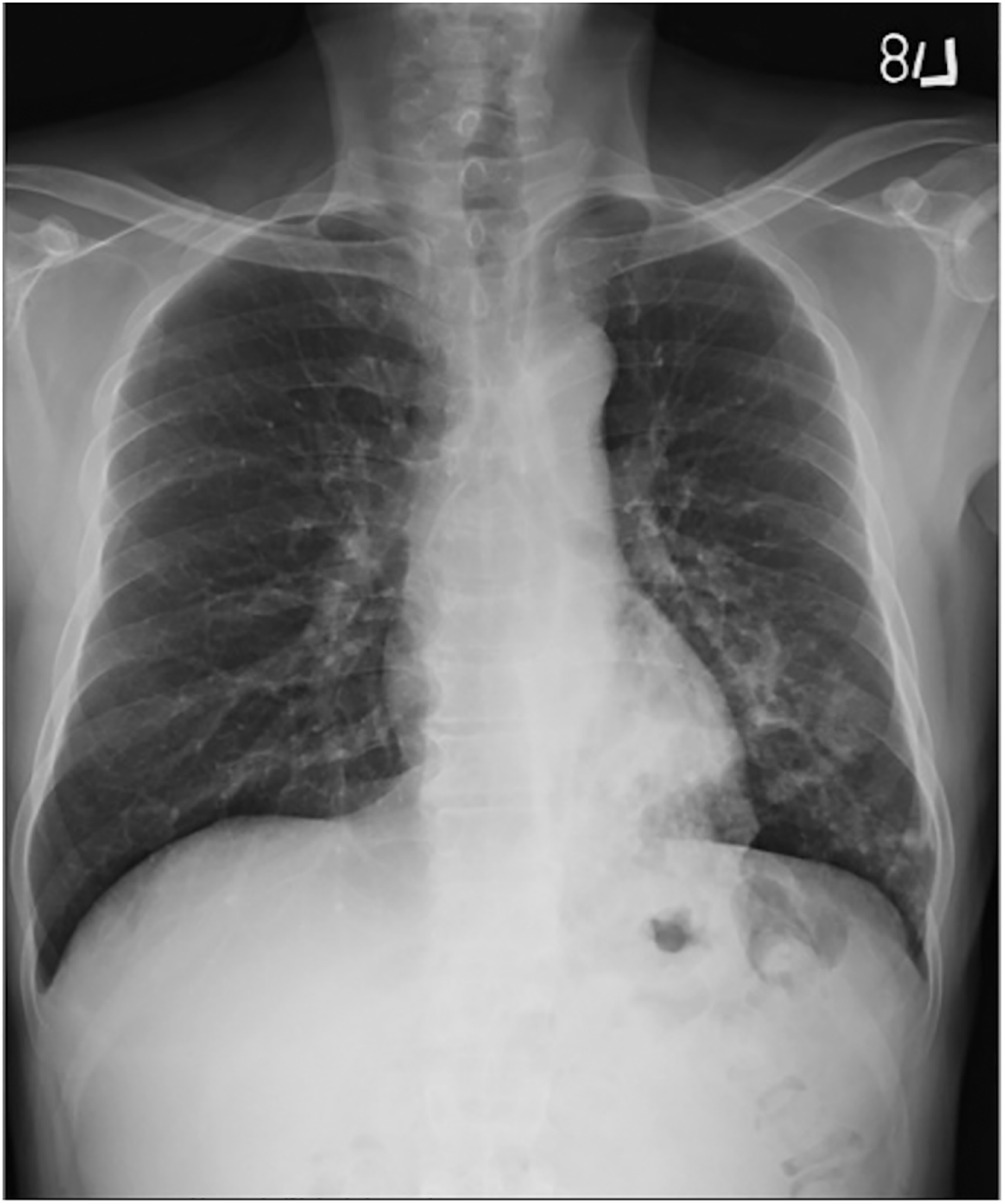
Primary pulmonary tuberculosis pattern with lower lung field lesions. A 53-year-old male with culture-proven pulmonary tuberculosis and a history of diabetes mellitus. The patient had a glycosylated hemoglobin (HbA1c) of 13.5%. CXR revealed patchy infiltrates and ill-defined acinar shadows in the left lower lung field.

**Fig 2:**
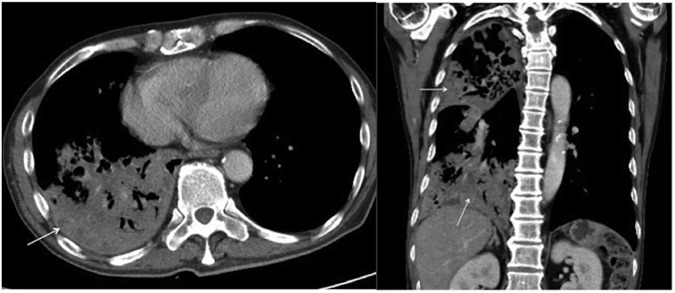
Primary pulmonary tuberculosis pattern with lobar consolidation. A 55-year-old male with culture-proven pulmonary tuberculosis and a history of diabetes mellitus. The patient had a glycosylated hemoglobin (HbA1c) of 9.5%. Axial (A) and coronal (B) contrast-enhanced thoracic CT scans revealed extensive consolidation (arrows) in the right upper and lower lobes.

**Fig 3:**
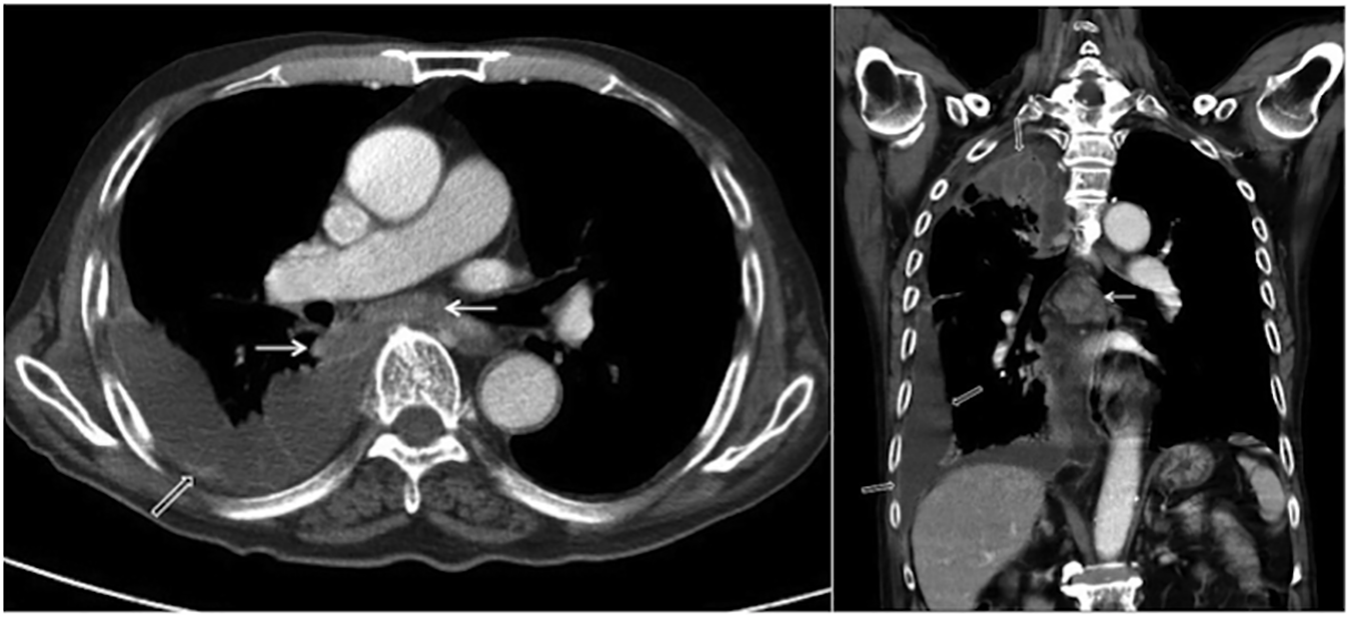
Primary pulmonary tuberculosis pattern with lymphadenopathy and pleural effusion. A 94-year-old male with culture-proven pulmonary tuberculosis and a history of diabetes mellitus. The patient had a glycosylated hemoglobin (HbA1c) of 9.4%. Axial (A) and coronal (B) contrast-enhanced thoracic CT scans revealed loculated right pleural effusion with pleural thickening (open arrows) and enlarged subcarinal and right interlobar lymph nodes with central low attenuation and peripheral rim enhancement (arrows).

Radiological manifestations of PTB in both groups are presented in Table 1. On CXRs findings, patients in TBDM group were significantly more likely to have unusual findings (*P* = 0.011) particularly in the primary TB pattern (*P* = 0.041) (Fig 1) and more extensive disease (moderately advanced and far advanced, *P* = 0.007 and 0.003, respectively) compared with those in TB group. Regarding thoracic CT manifestations, the findings indicative of primary PTB, including segmental or lobar consolidation (*P* = 0.003) (Fig 2), lymphadenopathy (*P* = 0.036) (Fig 3) and pleural effusion (*P* < 0.001) (Fig 3); other unusual findings, including large non-cavitary nodules/masses > 1 cm (*P* < 0.001) and more than one cavity in any single lesion (*P* < 0.001); predominant lesions in the unusual location (*P* = 0.016) and mixed location (*P* = 0.001); and diseases involving all lobes of lungs (*P* = 0.008) were more common in TBDM patients.

**Table 1:** Comparisons of demographics and radiological findings in diabetic and non-diabetic PTB patients.

Variable	DM (N = 214)	Non-DM (N = 214)	P value
**General demographics**			
Age, yr	72.5 ± 15.4 (25–98)	72.4 ± 15.6 (20–101)	0.935
Gender, male/female	172/42	172/42	1.000
Ever smoking (N, %)	102 (47.7%)	95 (44.4%)	0.561
**CXR (N = 214)**			
**Unusual findings** (N, %)	60 (28.0%)	37 (17.3%)	0.011
Primary TB^a^ (N, %)	46 (21.5%)	29 (13.6%)	0.041
Others^b^ (N, %)	14 (6.5%)	8 (3.7%)	0.274
** Extent**			
Minimal/Moderate/Far advanced^c^ (N, %)	78/116/20 (36.5%/54.2%/9.3%)	122/87/5 (57.0%/40.7%/2.3%)	<0.001
**CT (N = 123)**			
** Unusual findings**			
Primary TB			
Segmental or lobar consolidation (N, %)	59 (48.0%)	33 (26.8%)	0.003
LAP (short-axis diameter > 10mm) (N, %)	21 (17.1%)	9 (7.3%)	0.036
Pleural effusion (N, %)	37 (30.1%)	14 (11.4%)	< 0.001
Others			
Miliary lesions (N, %)	5 (4.1%)	5 (4.1%)	1.000
Pericardial effusion (N, %)	5 (4.1%)	1 (0.8%)	0.215
Large non-cavitary nodules/masses > 1cm (N, %)	35 (28.5%)	11 (8.9%)	< 0.001
> 1 cavity in one lesion (N, %)	26 (21.1%)	6 (4.9%)	< 0.001
** Location**			
Usual/Unusual/Mixed^d^ (N, %)	44/30/49 (35.8%/24.4%/39.8%)	86/14/23 (69.9%/11.4%/18.7%)	<0.001
** Distribution**			
All lobes involvement (N, %)	50 (40.7%)	28 (22.8%)	0.008

CT = computed tomography; CXR = chest radiograph; DM = diabetes mellidus; LAP = lymphadenopathy; TB = tuberculosis

^a^Lesions limited to lower lung fields (middle lobe, lingula and/or lower lobes), isolated hilar and/or mediastinal lymphadenopathy, pleural effusion alone, or more than one of the above findings.

^b^Miliary or disseminated lesions or negative finding.

^c^Minimal lesions = an area less than that above a horizontal line across the 2^nd^ chondrosternal conjunction of one lung; Moderately-advanced lesions = an area more than minimal lesions but less than one entire lung; Far advanced lesions = an area equivalent to or greater than one lung.

^d^Usual predominant = lesions limited to or predominantly involving the apical and posterior segments of the upper lobes and the superior segments of the lower lobes of lung; Unusual predominant = lesions limited to or predominantly involving the anterior segments of the upper lobes, the right middle lobe, lingular segment, and basal segments of the lower lobes of the lung; Mixed = even distribution of usual and unusual sites.

### Comparison of PTB in DM patients (TBDM group) with Hb_A1C_ ≤ 8% and those with Hb_A1C_ > 8%

The demographics and pretreatment CXRs findings of patients in the TBDM group divided by Hb_A1C_ of 8% were summarized in Table 2. In total, 101 cases (81 men and 20 women; age range, 29–97 years, mean ± SD 75.8 ± 14.5) had a pretreatment Hb_A1C_ ≤ 8% (range 5.2–8.0, mean 6.89 ± 0.74), and 113 cases (91 men and 22 women; age range 25–98 years, mean ± SD 69.5 ± 15.7) had a pretreatment Hb_A1C_ > 8% (range 8.1–16.4, mean 10.52 ± 2.01). Significant differences in Hb_A1C_ (*P* < 0.001) were noted between the two subgroups but not for age (*P* = 0.194) and gender (*P* = 1.000).

**Table 2:** Comparison of demographics and CXRs findings between diabetic PTB patients with HbA1c ≤ 8% and > 8%.

Variable	HbA1c ≤ 8% (N = 101)	HbA1C > 8% (N = 113)	P value
**Demographics**			
Age, yr	75.8 ± 14.5 (29–97)	69.5 ± 15.7 (25–98)	0.194
Gender, male/female (N, %)	81/20 (80.2%/19.8%)	91/22 (80.5%/19.5%)	1.000
Ever smoking (N, %)	48 (47.5%)	54 (47.8%)	1.000
HbA1c, %	6.89 ± 0.74 (5.2–8.0)	10.52 ± 2.01 (8.1–16.4)	< 0.001
**CXR**			
**Unusual findings** (N, %)	19 (18.8%)	41 (36.3%)	< 0.001
**Primary TB** (N, %)	18 (17.8%)	28 (24.8%)	0.246
Lower lung field TB alone (N, %)	11 (10.9%)	12 (10.6%)	1.000
LAP alone (N, %)	0 (0%)	0 (0%)	1.000
Pleural effusion alone (N, %)	1 (1.0%)	2 (1.8%)	1.000
> 1 of them (N, %)	6 (5.9%)	14 (12.4%)	0.157
**Others** (N, %)	1 (1.0%)	13 (11.5%)	< 0.001
Miliary lesions (N, %)	1 (1.0%)	5 (4.4%)	0.217
Disseminated lesions (N, %)	0 (0%)	6 (5.3%)	0.031
Negative (N, %)	0 (0%)	2 (1.8%)	0.499
**Usual findings** (N, %)	82 (81.2%)	72 (63.7%)	< 0.001
Fibronodular lesion without cavitation (N, %)	61 (60.4%)	51 (45.1%)	0.029
Patchy heterogeneous consolidation (N, %)	43 (42.6%)	49 (43.4%)	1.000
Bronchogenic spreading (N, %)	56 (55.4%)	67 (59.3%)	0.583
Tuberculoma (N, %)	8 (7.9%)	10 (8.8%)	1.000
**Extent**^**a**^			
Minimal (N, %)	49 (48.5%)	29 (25.7%)	< 0.001
Moderately advanced (N, %)	49 (48.5%)	67 (59.3%)	0.131
Far advanced (N, %)	3 (3.0%)	17 (15.0%)	<0.001

CXR = chest radiograph; HbA1_C_ = glycosylated hemoglobin; LAP = lymphadenopathy; TB = tuberculosis.

^a^ Minimal lesions = an area less than that above a horizontal line across the 2^nd^ chondrosternal conjunction of one lung; Moderately-advanced lesions = an area more than minimal lesions but less than one entire lung; Far advanced lesions = an area equivalent to or greater than one lung.

On CXRs manifestations, unusual findings (*P* < 0.001), other unusual findings (*P* < 0.001) in particular in disseminated lesions (*P* = 0.031), and far advanced extensive lesions (*P* < 0.001) are more common in patients with Hb_A1C_ > 8%. In contrast, the patients with Hb_A1C_ ≤ 8% were more likely to have a post-primary TB pattern (*P* < 0.001) and lesions with minimal extent (*P* < 0.001).

The subjects of the TBDM group with pretreatment thoracic CT scans were also divided in terms of Hb_A1C_. In total, 51 cases with Hb_A1C_ ≤ 8% and 72 cases with Hb_A1C_ > 8% were noted (Table 3). The Hb_A1C_ ≤ 8% subgroup (range 5.4–8.0, mean 6.89 ± 0.73) included 40 men and 11 women (age range 29–95 years, mean ± SD 72.5 ± 15.0), whereas the Hb_A1C_ > 8% (range 8.1–16.2, mean 10.61 ± 2.05) subgroup included 57 men and 15 women (age range 33–94 years, mean ± SD 68.8 ± 14.5). Significant differences in Hb_A1C_ (*P* < 0.001) were noted between the two subgroups but not for age (*P* = 0.581) and gender (*P* = 1.000).

**Table 3:** Comparison of demographics and CT findings between diabetic PTB patients with HbA1c ≤ 8% and > 8%.

Variable	HbA1c ≤ 8% (N = 51)	HbA1C > 8% (N = 72)	P value
**Demographics**			
Age, yr	72.5 ± 15.0 (29–95)	68.8 ± 14.5 (33–94)	0.581
Gender, male/female (N, %)	40/11 (78.4%/21.6%)	57/15 (79.2%/20.8%)	1.000
Ever smoking (N, %)	23 (45.1%)	31 (43.1%)	0.855
HbA1c, %	6.89 ± 0.73 (5.4–8.0)	10.61 ± 2.05 (8.1–16.2)	< 0.001
**CT**			
** Unusual findings**			
** Primary TB**			
Segmental or lobar consolidation (N, %)	21 (41.2%)	38 (52.8%)	0.271
LAP (short-axis diameter > 10mm) (N, %)	4 (7.8%)	17 (23.6%)	0.028
Pleural effusion (N, %)	13 (25.5%)	24 (33.3%)	0.426
** Others**			
Miliary lesions (N, %)	0 (0%)	5 (6.9%)	0.076
Pericardial effusion (N, %)	1 (2.0%)	4 (5.6%)	0.402
Large non-cavitary nodules/masses > 1cm (N, %)	10 (19.6%)	25 (34.7%)	0.072
> 1 cavity in one lesion (N, %)	3 (5.9%)	23 (31.9%)	< 0.001
** Usual findings**			
Centrilobular nodules (N, %)	21 (41.2%)	23 (31.9%)	0.342
Tree-in-bud opacities (N, %)	26 (51.0%)	43 (59.7%)	0.057
Single cavitary nodule/mass (N, %)	13 (25.5%)	23 (31.9%)	0.547
Satellite nodules (N, %)	17 (33.3%)	34 (47.2%)	0.140
Acinar or lobular nodules (N, %)	32 (62.7%)	56 (77.8%)	0.104
** Location**			
Usual predominant^a^ (N, %)	23 (45.1%)	21 (29.2%)	0.087
Unusual predominant^b^ (N, %)	11 (21.6%)	19 (26.4%)	0.671
Mixed^c^ (N, %)	17 (33.3%)	32 (44.4%)	0.263
** Distribution**			
All lobes involvement (N, %)	15 (29.4%)	35 (48.6%)	0.041

CT = computed tomography; HbA1_C_ = glycosylated hemoglobin; LAP = lymphadenopathy; TB = tuberculosis.

^a^Lesions limited to or predominantly involving the apical and posterior segments of the upper lobes and the superior segments of the lower lobes of lung.

^b^Lesions limited to or predominantly involving the anterior segments of the upper lobes, the right middle lobe, lingular segment, and basal segments of the lower lobes of the lung.

^c^Even distribution of usual and unusual sites.

On thoracic CT manifestations, patients with Hb_A1C_ > 8% were more likely to have unusual findings of lymphadenopathy (*P* = 0.028), more than one cavity in any single lesion (*P* < 0.001) and disease involving all lung lobes (*P* = 0.041).

## Discussion

Consistent with the results of previous reports [8–13,17,26], our results demonstrated that unusual manifestations on CXRs were more frequently noted in diabetics compared with non-diabetics. An increased frequency of unusual findings and unusual location of disease on thoracic CT scans were also noted in diabetic PTB patients. Therefore, diabetes may have potential effects on the radiological manifestations of PTB. Moreover, we found that diabetic patients had more extensive PTB disease on both CXRs and thoracic CT scans. Given the increased frequency of atypical radiological findings and unusual location of PTB lesions in our results, the relevant difficulty in image interpretation leading to a delay of diagnosis by clinicians may partly contribute to the more extensive lung involvement of PTB in diabetic subjects.

The mechanisms underlying atypical image findings of PTB in diabetic patients remain unclear. Immune derangements (or dysfunctions) were identified in the studies of both hyperglycemic mice [27,28] and diabetic individuals [29–31], which might play a vital role in their TB susceptibility. The predominant abnormalities affect the cell-mediated arm of the immune system. However, there was no definite correlation of dysfunctional immune process and radiological manifestations of PTB. In the study of Perez-Guzman et al [8], although a reduced number of non-lymphocyte leukocytes were observed in diabetic PTB patients, it did not statistically contribute to the atypical radiological images. Additionally, no difference of lymphocyte counts in peripheral blood was noted between diabetic and non-diabetic TB subjects. In terms of pulmonary physiology, Perez-Guzman and colleagues reported an increased frequency of lower lung lesions in elderly non-diabetic TB patients and in all ages of diabetic TB subjects [26]. Additionally, similar structural modifications of alveoli and pulmonary microvasculature were observed in the aging process and diabetes [32]. Accordingly, they postulated that similar physiological dysfunction with an increased ventilation/perfusion (V/Q) ratio and a consequent increased alveolar oxygen pressure (PAO_2_) might predispose the growth of *Mycobacterium tuberculosis* in the lower lung fields of diabetic patients. However, in a small sample size study of ventilation-perfusion scintigraphy among diabetics exhibiting a high V/Q ratio in 40% of the patients (8 out of 20), only one case had this physiological change in the lower lobes [33]. Therefore, the correlation between pulmonary physiological dysfunction and atypical lower lung field TB in diabetics deserves verification through further studies.

Our study also explored the correlation between glycemic status and radiological manifestations of PTB in diabetic patients. Individuals of poor glycemic control with Hb_A1C_ > 8% were more likely to have unusual CXRs findings and more advanced disease. In contrast, the post-primary TB pattern was more frequent in those with Hb_A1C_ ≤ 8%. Our results were consistent with the findings of Chiang’s report [17]. Regarding thoracic CT manifestations, a statistically significant higher prevalence of unusual findings and lesions involving all lobes of the lung were noted in diabetics with Hb_A1C_ > 8%. Accordingly, glycemic status has a potential effect on radiological manifestations and disease extent in diabetic PTB patients.

In the 41 diabetic PTB patients with Hb_A1C_ > 8% exhibiting unusual CXRs findings, 11 of them received thoracic CT scans (Table 4). However, although the thoracic CT images were expected to provide more detailed and thorough diagnostic information, a straightforward diagnosis of PTB could only be made in four (cases 1, 2, 8, and 10) of the 11 patients by the reading physicians. Therefore, as a possibly limited role of thoracic CT scans in aiding the diagnosis of PTB in poorly controlled diabetic patients, a high index of suspicion with early clinical work-ups are absolutely critical for PTB detection in these subjects.

**Table 4:** Demographics and CT findings in diabetic PTB patients with HbA1c > 8% having unusual CXR pattern.

Case No.	Age	Gender	HbA1c, %	CXR findings	CT findings
1	89	male	12.9	Disseminated ill-defined nodules and consolidative patches, cavitary lesions	Cavitary lesions in RB1/RB2, LB1+2, RB6 (1 or > 1 cavity in single lesion), noncavitary nodules, acinar opacities, TIB infiltrates, right PE, pericardial effusion; mixed location
2	92	male	11.1	Miliary nodules	Miliary nodules, TIB, acinar nodules; mixed location
3	66	male	11.2	Bilateral lower lung field consolidation, left PE	Consolidative patches, left pleural effusion; unusual location
4	33	male	16.2	Left lower lung field consolidation	LLL lobar consolidation, acinar nodules; unusual location
5	55	male	9.4	Consolidative patches and ill-defined nodules in right upper and lower lung fields, right PE	RUL/RML/RLL lobar consolidation, bilateral acinar nodules/TIB, cavitation in RB1/LB3, mediastinal LAP, right loculated PE; mixed location
6	48	female	10.6	Lower lung consolidation, right PE	Lobar or segment consolidation in RUL/RML/RLL, right loculated PE, TIB in LB1+2; mixed location
7	75	female	14.8	Bilateral lower lung infiltrates	Bilateral TIB and acinar opacities, cavitary lesions; mixed location
8	37	male	11.6	Right upper and bilateral lower lung consolidation, right PE	Segmental consolidation in RUL, lingula & RLL, cavitations (1 or > 1 cavity in single lesion), acinar shadow and TIB in LB1+2/LB3, right PE; mixed location
9	87	male	9.4	Bilateral PE	Bilateral PE, mediastinal LAP; unusual location
10	94	female	10.9	Right lower lung patches, right PE	Centrilobular nodules, TIB, acinar nodules, and satellite lesions in LB1+2 and RB6/RB9/RB10, right PE; usual location
11	53	male	13.5	Left lower lung consolidation	Consolidation and acinar nodules in LLL, unusual location

CT, computed tomography; CXR, chest radiograph; HbA1_C_, glycosylated hemoglobin; LAP, lymphadenopathy; LB1+2, apicoposterior segment of left upper lobe; LB3, anterior segment of left upper lone; LLL, left lower lobe; PE, pleural effusion; RB1, apical segment of right upper lobe; RB2, posterior segment of right upper lobe; RB3, anterior segment of right upper lobe; RB6, superior segment of right lower lobe; RB9, lateral basal segment of right lower lobe; RB10, posterior basal segment of right lower lobe; RLL, right lower lobe; RML, right middle lobe; RUL, right upper lobe; TB, tuberculosis; TIB, tree-in-bud.

The study had some limitations. First, the retrospective nature and limited patient sample size in the current study may introduce a possible selection bias, which potentially limited our conclusions. Second, the image protocol could not be uniformly implemented in all cases due to the retrospective study design, especially in the thoracic CT scans. Third, the results of our findings may not be generalized to the areas or countries with non-endemic TB. Fourth, although our study demonstrated a possibly limited role of the thoracic CT scans in aiding the diagnosis of PTB in poorly controlled diabetic patients, a small sample size may not achieve a solid conclusion. Further studies with larger study populations are needed to clarify this issue. Finally, *Mycobacterium tuberculosis* strains were not studied. Our previous work indicated that *Mycobacterium tuberculosis* strains might affect the radiological presentation of PTB [34]. However, our results may still have clinical relevance. Given the increased frequency of unusual radiological findings and extensive disease on CXRs and thoracic CT scans of PTB observed in diabetics, those without appropriate glycemic control may have an even higher risk. Therefore, when interpreting chest images, radiologists and chest physicians should pay more attention to diabetic patients with poor blood sugar control to avoid underdiagnoses of PTB.

## Conclusions

Unusual radiological patterns with atypical locations and extensive PTB disease were more frequently observed in diabetics. Among them, subjects with poor blood sugar control exhibit a higher risk. Awareness of these unusual radiological features of PTB on CXRs and thoracic CT scans in the diabetic patients as well as a high index of suspicion in those with poor glycemic control are of considerable value in establishing an early diagnosis of PTB and avoiding treatment delays. Further studies are needed to clarify these issues.

## Supporting information

S1 Table Table 1 Data. **Raw data of TBDM group.** Excel table containing gender, age, smoking status, HbA1c level, and CXR and thoracic CT scan findings of diabetic PTB patients (TBDM group).
(XLSX)

S2 Table Table 1 Data. **Raw data of TB group**. Excel table containing gender, age, smoking status, and CXR and thoracic CT scan findings of PTB patients without DM (TB group).
(XLSX)

S3 Table Table 2 Data. **Raw data**. Excel table containing gender, age, smoking status, HbA1c level, and CXR findings of diabetic PTB patients dividing into controlled with HbA1C < 8% and uncontrolled with HbA1C > 8%.
(XLSX)

S4 Table Table 3 Data. **Raw data.** Excel table containing gender, age, smoking status, HbA1c level, and thoracic CT scan findings of diabetic PTB patients dividing into controlled with HbA1C < 8% and uncontrolled with HbA1C > 8%.
(XLSX)
